# HPLC, NMR Based Characterization, Antioxidant and Anticancer Activities of Chemical Constituents from Therapeutically Active Fungal Endophytes

**DOI:** 10.4014/jmb.2403.03036

**Published:** 2024-05-06

**Authors:** Waqas Hussain Shah, Wajiha Khan, Sobia Nisa, Michael H.J. Barfuss, Johann Schinnerl, Markus Bacher, Karin Valant-Vetschera, Ashraf Ali, Hiba-Allah Nafidi, Yousef A. Bin Jardan, John P. Giesy

**Affiliations:** 1 Department of Biotechnology, COMSATS University Islamabad, Abbottabad Campus; 2 Department of Microbiology, The University of Haripur, Haripur 22620, Pakistan; 3 Department of Botany and Biodiversity Research, University of Vienna, Rennweg 14, 1030 Vienna, Austria; 4 Department of Chemistry, Institute of Chemistry of Renewable Resources, University of Natural Resources and Life Sciences (BOKU), Tulln 3430, Austria; 5 Department of Chemistry, Faculty of Physical and Applied Sciences, The University of Haripur, Haripur 22620, Pakistan; 6 School of Chemistry and Chemical Engineering, Henan University of Technology, Zhengzhou 450001, P.R. China; 7 Department of Food Science, Faculty of Agricultural and Food Sciences, Laval University, 2325 Quebec City, QC G1V 0A6, Canada; 8 Department of Pharmaceutics, College of Pharmacy, King Saud University, P.O. Box 11451, Riyadh, Saudi Arabia; 9 Toxicology Centre, University of Saskatchewan, Saskatoon, SK S7N 5B3, Canada; 10 Department of Veterinary Biomedical Sciences, University of Saskatchewan, Saskatoon, SK S7N 5B4, Canada; 11 Department of Integrative Biology, Michigan State University, East Lansing, MI 48824, USA; 12 Department of Environmental Sciences, Baylor University, Waco, TX 76706, USA; 13 Department of Clinical Laboratory Sciences, College of Applied Medical Sciences, King Saud University, P.O. Box 10219, Riyah, 11433, Saudi Arabia

**Keywords:** Endophytic fungi, bioactive compounds, *Silybum marianum*, *Aspergillus*, antibacterial activity, antibiofilm activity

## Abstract

Fungi generate different metabolites some of which are intrinsically bioactive and could therefore serve as templates for drug development. In the current study, six endophytic fungi namely *Aspergillus flavus*, *Aspergillus tubigenesis*, *Aspergillus oryzae*, *Penicillium oxalicum*, *Aspergillus niger*, and *Aspergillus brasiliensis* were isolated and identified from the medicinal plant, *Silybum marianum*. These endophytic fungi were identified through intra transcribed sequence (ITS) gene sequencing. The bioactive potentials of fungal extracts were investigated using several bioassays such as antibacterial activity by well-diffusion, MIC, MBC, anti-biofilm, antioxidant, and haemolysis. The *Pseudomonas aeruginosa* PAO1 was used to determine the antibiofilm activity. The ethyl acetate extract of *Aspergillus flavus* showed strong to moderate efficacy against *Staphylococcus aureus*, *Escherichia coli*, *P. aeruginosa*, and *Bacillus spizizenii*. *Aspergillus flavus* and *Aspergillus brasiliensis* exhibited significant antibiofilm activity with IC_50_ at 4.02 and 3.63 mg/ml, while *A. flavus* exhibited maximum antioxidant activity of 50.8%. Based on HPLC, LC-MS, and NMR experiments kojic acid (1) and carbamic acid (methylene-4, 1-phenylene) bis-dimethyl ester (2) were identified from *A. flavus*. Kojic acid exhibited DPPH free radical scavenging activity with an IC_50_ value of 99.3 μg/ml and moderate activity against ovarian teratocarcinoma (CH1), colon carcinoma (SW480), and non-small cell lung cancer (A549) cell lines. These findings suggest that endophytic fungi are able to produce promising bioactive compounds which deserve further investigation.

## Introduction

Antibiotic resistance is rapidly spreading around the world and the situation is rapidly deteriorating, rendering current antimicrobial medications ineffective [1-5]. Each year, antibiotic-resistant bacteria infect approximately two million people worldwide, resulting in at least 23,000 deaths [6-8]. Furthermore, considering the various problems and side effects connected with existing antimicrobial drugs, it is not unexpected that a significant number of people are turning to alternative treatments. Approximately 80% of the population in developing nations, primarily in Africa, Asia, and Latin America, depends on plants with medicinal properties to fulfill their basic healthcare and wellness requirements [9-12].The use of plants dates back a long time as a source of therapeutic chemicals to treat diseases [13]. The current use of antibiotics and anticancer compounds has led to decreased effectiveness, particularly with potentially lethal bacterial biofilms [14, 15]. To address this issue, the discovery of novel compounds from sources such as endophytic fungi have been useful [16, 17]. The study of endophytes is a rapidly growing field, with increased scientific attention and interest due to advancements in characterization and applications of natural products in biological control and crop production [18]. Fungal endophytes are a large reservoir of bioactive compounds with capability of drugs useful for treatment of a variety of health problems [19-21]. Novel anticancer drugs, antibacterial agents, substances that suppress the immune system, volatile antibiotic combinations, antioxidants, and hydrocarbons are among these substances. The field offers opportunities for scientific discovery and innovation across multiple important areas [22-25].

*Silybum marianum* (L.) Gaertn., (Asteraceae) commonly known as milk thistle is a native plant of Northern Africa, Asia, Southern Russia, and Southern Europe [26, 27]. Milk thistle is largely utilized in gastrointestinal clinics to treat cirrhosis and hepatitis [28]. From this plant species flavonolignans like silibinin A and B and flavanonols like taxifolin are known [29]. Previous research on this plant revealed a variety of endophytic fungi producing a broad array of metabolites [30].

The goal of this research was to isolate, identify, and assess antimicrobial activities of endophytic fungi found in the milk thistle collected in Pakistan. In total, twelve endophytic fungi strains were isolated from *S. marianum*. Among them, six endophytic fungi were identified through ITS gene sequencing. Various bioassays were used to evaluate the bioactive potential ethyl acetate extracts of fungi (antibacterial activity by well-diffusion, MIC, MBC, anti-biofilm, antioxidant, and hemolysis).

## Materials and Methods

Endophytic fungi were isolated from *S. marianum* collected from Haripur (Latitude 33.7333 and longitude 72.5833) between January to April 2020 and identified by experts at plant taxonomy lab, Quaid-e-Azam University Islamabad, Pakistan. A voucher specimen was deposited in the Quaid-e-Azam University herbarium with the specimen number ISL-182311.

### Sample Collection, Surface Sterilization, and Culturing of Endophytic Fungi

Various samples of the milk thistle were collected from plants that exhibited healthy characteristics. Plant parts, leaves, stem, and fruits were collected separately. Following collection, the plant components were stored in polythene bags under conditions of high humidity. Subsequently, plant materials were cut into 5 mm sections. The plant materials were sterilized by submerging them subsequently in ethanol (70%) and sodium hypochlorite (2%) each for 1 min, as previously described [31]. Before plating, the plant materials were kept under sterile conditions for air drying. In order to prevent bacterial contamination, segments of plants were placed on potato dextrose agar (PDA) plates containing 50 μg/ml of chloramphenicol. The plates were then sealed with parafilm and kept in a Biological Oxygen Demand (BOD) incubator set to a temperature of 28 ± 2°C for a period of 5–8 days. Using sterile techniques, fungal colonies were transferred from hyphal tips onto sterile PDA plates to produce pure cultures of the fungi. Fungi differing in morphology were selected for further studies [32].

### Fermentation of Endophytic Fungi

For the production of secondary metabolites from endophytes, the fungi were grown on PDA. The culture medium was adjusted to pH 5.6 before autoclaving, and then 100 ml of sterile medium was inoculated with the isolated fungus and incubated in a shaking incubator at 28°C and 170 rpm for 4 weeks [33]. The mycelium was removed from the medium and the solute was filtered and treated with ethyl acetate to extract potentially bioactive compounds. Using a rotary evaporator, the ethyl acetate extract was concentrated at 35°C and mass of extract was recorded [34].

### Determination of Antibacterial Activity

To evaluate antibacterial potential of crude extracts from cultured endophytic fungi, the well-diffusion method was employed on Gram-negative and Gram-positive bacterial strains. On a Muller Hinton Agar (MHA) plate, the pathogenic bacterial culture was spread out and wells were created with a borer. These wells were then treated with three concentrations of extract (10, 5 or 1 mg/ml). After overnight incubation at 37°C, a noticeable zone of inhibition was seen around each well [31]. Ciprofloxacin was used as positive control while DMSO acted as the negative control.

### Determination of IC_50_ and Minimum Bactericidal Concentration

To determine the IC_50_ Value of the active extracts, a two-fold serial dilution was done in Cation Adjusted Mueller Hinton Broth (CAMHB) Medium. To reach a final bacterial culture size of 106 CFU/ml, the diluted extract was added to 96-well microtiter plates and allowed to incubate overnight. After overnight incubation at 37°C, the microtiter plate reader was utilized to determine the optical density, with a wavelength of 600 nm. The IC_50_ value was then computed with the help of the Easy IC_50_ Calculation Tool Kit (http://www.ic50.tk). To determine the minimum bactericidal concentration (MBC), the experimental mixture was cultured for another 24 h at 37°C, and the number of colonies (CFU/ml) was counted. The MBC of the extract was considered the concentration that reduced the count by ≥ 90% compared to the control cultures (untreated) [35]. All tests were performed in triplicates.

### Radical Scavenging Activity

The purified compounds 1 and 2 were subjected to antioxidant activity by DPPH free radical scavenging assay using a 96-well plate. The compounds were dissolved in methanol and dilution series from 0.2 to 500 μg/ml reaching a total volume of 50 μl in each well were prepared. Subsequently, freshly prepared 50 μl DPPH solution at a conc of 0.004% was added and the absorbance at 517 nm measured after 30 min using the Tecan Infinite M Nano measured. The EC_50_ value were calculated using the online tool from www.ic50.tk. Ascorbic acid served as positive control [36].

### Determination of Biofilm Inhibition

In a 1:100 ratio, a diluted overnight culture was inoculated into tryptic soy broth and allowed to grow to reach its exponential phase, which typically occurs within 5 h. The turbidity of the culture was then checked to assure that it was at least 0.5 McFarland units. In a 96-well plate, increasing concentrations of the extracts were added to the culture without shaking, and incubated for 24 h at 37°C. Following the incubation, the non-adherent cells were separated by washing the wells twice with Phosphate-Buffered Saline (200 μl). The biofilm-coated cells were fixed using 100% methanol (200 μl), and then stained with Crystal Violet (200 μl) for a duration of 20 min. After discarding the surplus stain with PBS, the plates were allowed to air-dry. The stained crystal violet was extracted using 33% acetic acid (200 μl). To evaluate the inhibition of biofilms, a microtiter plate reader (add the type and the company of this instrument) was utilized to measure the absorbance at OD 595 nm, and the inhibition percentage was computed.

### Determination of Biocompatibility

To evaluate the biocompatibility of the fungal extract the hemolysis assay was employed. Sheep blood (10 ml) was centrifuged at 2,400 ×*g* for 300 s, and then washed it two times with PBS. Extract added to the pellet, incubated for 60 min at 37°C, and centrifuged again for 300 s at 2,400 g. The wavelength of 540 nm was used to measure the optical density of the supernatant using a spectrophotometer, with water and erythrocyte suspension as the blank. A positive control was established using erythrocyte solution and 1% Triton X-100 [37].

### Molecular Identification

DNA extraction was from nine bioactive fungi by using the modified chloroform/phenol method based on bioassay results [38]. Fungal mycelium, which had been briefly lyophilized, was crushed with extraction buffer using a pestle and mortar. The ground samples were then incubated for 30 min at 65°C in a 1.5 ml Eppendorf tube. After cooling, the tubes were centrifuged at 11,000 g for 10 min to collect the aqueous phase in a new tube. A solution of phenol, chloroform, and isoamyl alcohol in a ratio of 24:25:1 was added to an equal amount of supernatant, mixed by vortexed, and then centrifuged on same condition. The aqueous layer was decanted in a sterile tube and same amount of absolute ethanol was used to precipitate the DNA. Washing of precipitated DNA was done by chilled 70% ethanol. 40 μl TE solutions was added and stored at -20°C.

For molecular identification the nuclear ribosomal ITS region was amplified and sequenced using the primers ITS5 (forward, 5'-GGA AGT AAA AGT CGT AAC AAG G-3') and ITS4 (reverse, 5'-TCC TCC GCT TAT TGA TAT GC-3') [39]. The PCR reaction mix contained in a final reaction volume (10 μl): (1) 5 μL 2 × Phusion Plus Green PCR Master Mix (Thermo Fisher Scientific, USA); (2) 1 μl of 5 μM each primer (Sigma-Aldrich, USA); (3) 0.1 μl of 20 mg/ml BSA (Thermo Fisher Scientific); (4) 1.9 μl of 1 M Trehalose (Sigma-Aldrich); and (5) 1 μl of diluted DNA template. The PCR reactions were performed in an Eppendorf Mastercycler (Eppendorf, Austria) with the following cycling parameters: 1 cycle with 98°C for 30 s; 35 cycles each with 98°C for 10 s, 55°C for 20 s, 72°C for 30 s; 1 cycle with 72°C for 5 min; and a final hold at 8°C. PCR products were purified with a 1:2 mixture of Exonuclease I (20 U/μl; Thermo Fisher Scientific) and FastAP Thermosensitive Alkaline Phosphatase (1 U/μl; Thermo Fisher Scientific) according to [40]. We added 1 μl of the enzyme mixture to each 9 μl PCR reaction (after verifying 1 μl on an agarose gel) and incubated at 37°C for 30 min, followed by a deactivation of the enzymes at 85°C for 15 min. Cycle sequencing reactions were performed on a 96-Well GeneAmp PCR System 9700 (Thermo Fisher Scientific) according to the BigDye Terminator v3.1 Cycle Sequencing Kit (Thermo Fisher Scientific) using PCR primers. We used slightly modified PCR conditions: 1 cycle with 96°C for 1 min; 35 cycles each with 96°C for 10 s, 50°C for 5 s; 60°C for 3 min; and a final hold at 4°C. Reaction components were: 0.6 μl of BigDye Terminator v3.1, 1 μl of 5 μM primer, 1.7 μl of 5× sequencing buffer (Thermo Fisher Scientific), 2 μl of 1 M Trehalose, 2 μl of purified PCR product, and 2.7 μl of PCR-grade water. Sephadex-cleaned products were run on a 3730 DNA Analyzer (Thermo Fisher Scientific) following manufacturer’s instructions.

### Phylogenetic Analyses

Sequences were assembled and edited using SeqMan Pro (Lasergene 8.1, DNASTAR, USA), and the consensus was exported in fasta format and deposited in Gene Bank (Table 5). Sequences similar to isolated fungal endophytes were collected from the NCBI. These sequences were then aligned using the Clustal W program, which is a widely used software for multiple sequence alignment. The result of the alignment process was saved in MEGA format, which is a commonly used file format for storing multiple sequence alignments. After alignment was completed, phylogenetic trees were constructed to visualize the evolutionary relationships among the fungal endophytes, using the neighbor-joining method in MEGAX, which is software for performing evolutionary analyses. The parameters used in the analysis were set to default, which means that the software used its built-in settings. In addition, 1000 bootstrap values were used to assess the confidence of the relationships depicted in the tree. Bootstrapping is a statistical method that involves resampling the data many times to estimate the reliability of the results [41, 42].

### Analytical Procedures

Thin layer chromatography (TLC) was performed on precoated silica gel 60 _F254_ aluminia foil plates. The plates were developed in *n*-heptane, ethyl acetate, and methanol in a ratio of 70:25:5 and visualized under a UV lamp at 254 nm. The plates were subsequently sprayed with anisaldehyde reagent. HPLC analyses were performed on Agilent 1100 Series with UV-diode array detector (DAD) detector, Hypersil BDS-C18 column (250 × 4.6 mm, 5 μm particle size), eluted with MeOH in aqueous buffer (15 mM ortho-H_3_PO_4_ and 1.5 mM tetrabutyl ammonium hydroxide) with a flow rate of 1.0 ml/min, injection volume of 10 μl and linear gradient starting from 20 to 90%MeOH at 17 min to 100% at 20 min for 8 min. The detection wavelength was adjusted at 230 nm (reference WL 360 nm).

LC-MS analyses were conducted to produce mass spectra of isolated compounds. Direct infusion electrospray ionization (ESI) was used to obtain mass spectra between *m/z* 100–2500 of purified compounds. Capillary voltage 4 kV with a capillary current of 30–50 nA and nitrogen temperature 180°C was applied with a flow rate of 4.0 L/min and the N2 nebulizer gas pressure at 0.3 bar [43].

Nuclear magnetic resonance NMR spectra for structure elucidation were acquired on a Bruker Avance III 600 spectrometer equipped with a 5 mm N_2_ cooled cryo probe head (Prodigy, USA) with standard Bruker pulse programs. Chemical shifts are given in ppm referenced to residual solvent signal (MeOD: δ_H_ 3.31 and δ_C_ 49.0 ppm, CDCl3: δ_H_ 7.26 and δ_C_ 77.0 ppm).

### Isolation of Compounds 1 and 2

Column chromatography over silica gel 60 (40–63 μm grain size) was performed for crude ethyl acetate extract (800 mg) of SMS6, and a total of 15 fractions a 50 ml were collected using mixtures of *n*-heptane, ethyl acetate and methanol in different combinations as mobile phase starting with 100% *n*-heptane. A total of 15 fractions were collected and the solvent evaporated. All the obtained fractions were analyzed by TLC and HPLC. The fractions having similar HPLC profiles were merged (52.3 mg) and further purified by size exclusion chromatography over Sephadex LH20 (GE Healthcare, Germany) as stationary phase eluted with methanol. The approximate volume of each collected fraction was 2.5 ml. Fraction 9 yielded compound 1 (2.3 mg), while fractions 10–13 contained 1.7 mg of compound 2.

### Antibacterial Activity

Different concentrations (0.015–2 μg/ml) of kojic acid (1) and carbamic acid, (methylene-4, 1-phenylene) bis-dimethyl ester (2) were used to determine antibacterial activity against *Lactobacillus lactis* and *E. coli* by disk diffusion method. Ampicillin was used as positive control and zones of inhibition were measured in mm.

### Anticancer Activity

The MTT (3-(4,5-dimethylthiazol-2-yl)-2,5-diphenyltetrazolium bromide) assay was used to determine cytotoxicity of compound that indicated DPPH free radical scavenging assay against Ovarian Teratocarcinoma (CH1), Colon Carcinoma (SW480), and Non-small Cell Lung Cancer (A549) Cell Lines. CH1/PA-1 cells (identified via STR profiling as PA-1 ovarian teratocarcinoma cells by Multiplexion, Germany) were a gift from Lloyd R. Kelland, CRC Center for Cancer Therapeutics, Institute of Cancer Research, Sutton, UK. SW480 (human adenocarcinoma of the colon) and A549 (human non-small cell lung cancer) cells were provided by the Institute of Cancer Research, Department of Medicine I, Medical University of Vienna, Austria. All cell culture media, supplements and assay reagents were purchased from Sigma-Aldrich, and plasticware from Starlab.

Cells were grown in 75 cm^2^ culture flasks as adherent cultures in minimum essential medium (MEM) supplemented with 10% heat inactivated fetal bovine serum (FBS; BioWest, France), 1 mM sodium pyruvate, 4 mM L-glutamine, and 1% non-essential amino acids (from a 100 × ready-to-use stock). Cultures were maintained at 37°C in a humidified atmosphere containing 5% CO_2_. Subconfluent SW480 (colon carcinoma), CH1/PA-1 (ovarian teratocarcinoma) and A549 (non-small cell lung cancer) cells were harvested by trypsinization for 3–5 min at 37°C in a humidified atmosphere. Supplemented MEM was added to stop trypsinization, and cells were centrifuged for 3 min at 1,200 rpm (Thermo Scientific, Megafuge 1.0R). After aspiration of the supernatant, the cell pellet was resuspended in supplemented MEM. Afterwards, CH1/PA-1, SW480 and A549 cells were seeded in 100 μl aliquots in densities of 1.0 × 10^3^, 2.0 × 10^3^, and 3.0 × 10^3^ cells/well, respectively, in clear flat-bottom 96-well microculture plates. After incubation of the plates for 24 h, the test compound was dissolved in 100% DMSO, serially diluted in supplemented MEM and added in triplicates of 100 μL/well, whereupon the concentration of DMSO did not exceed 0.5% v/v. Plates were incubated for 96 h, then the medium was replaced with 100 μl/well of an MTT-medium mixture. For this purpose, MTT powder had been dissolved in PBS to a concentration of 5 mg/ml and then diluted 1:7 in supplemented RPMI 1640 medium (supplemented with 10% heat-inactivated FBS and 4 mM L-glutamine). After 4 h of incubation, the dyeing solution was replaced with 150 μl/well of DMSO and optical densities were measured at 550 nm (with 690 nm as reference) with a microplate reader (BioTek, ELx808, USA). Interpolated IC_50_ values were averaged from at least three independent experiments.

### Statistical Analysis

All the measures were performed in triplicate. Results were calculated for normal percentage inhibition whereas the information gotten from activity of division was statistically analyzed by ANOVA and Man-Whitney U test using MSTATC. Prior to the use of parametric statistical procedures, you should check for normality by using the Shapiro–Wilks test and the assumption of homogeneity of variance should be evaluated using Levene’s test. For concentration data that are not normally distributed, the data can be transformed using the natural log (ln) of (x + 1) and then parametric statistics applied or you can apply the appropriate non-parametric statistics, such as the Man-Whitney U test or Wilcoxin’s test. The non-parametric tests are performed on the ranks instead of the actual values. Non-parametric tests are less powerful than the parametric tests, so if possible use the parametric tests. I generally apply both parametric and non-parametric tests and come pare the result to determine the robustness of the statistics. If both give the same result then there is no issue. But if the non parametric do not show a difference there may be insufficient power. You should always estimate the power of the test before doing a study so that you can determine the necessary sample size. Also, it is important to know the experimental unit of the study and not have issues with pseudo-replication. Check out these concepts on-line and get in touch if you have questions or need help.

## Results

In total, twelve fungal strains were isolated from *S. marianum*, six fungal strains from leaves, five from the stem and one from the fruit. Sequencing of ITS led to the identification of the strains namely *A. flavus* (SML 2.1), *Aspergillus tubigenesis* (SML 2.2), *Aspergillus flavus* (SML3), *Aspergillus oryzae* (SML5), *Penicillium oxalicum* (SML6.2) were isolated from leaves, *Aspergillus niger* (SMS1) from stem and *Aspergillus brasiliensis* (SMF2) from fruits. From extracts of the identified strains various bioactivites were assessed (see the subsequent sections). Additionally, to the identificiation of the fungi, two compounds from SMS 6 namely kojic acid (1) and carbamic acid (methylene-4, 1-phenylene) bis-dimethyl ester (2) could be isolated and identified.

### Antibacterial Activity of Fungal Endophytes

**Fig. 1:**
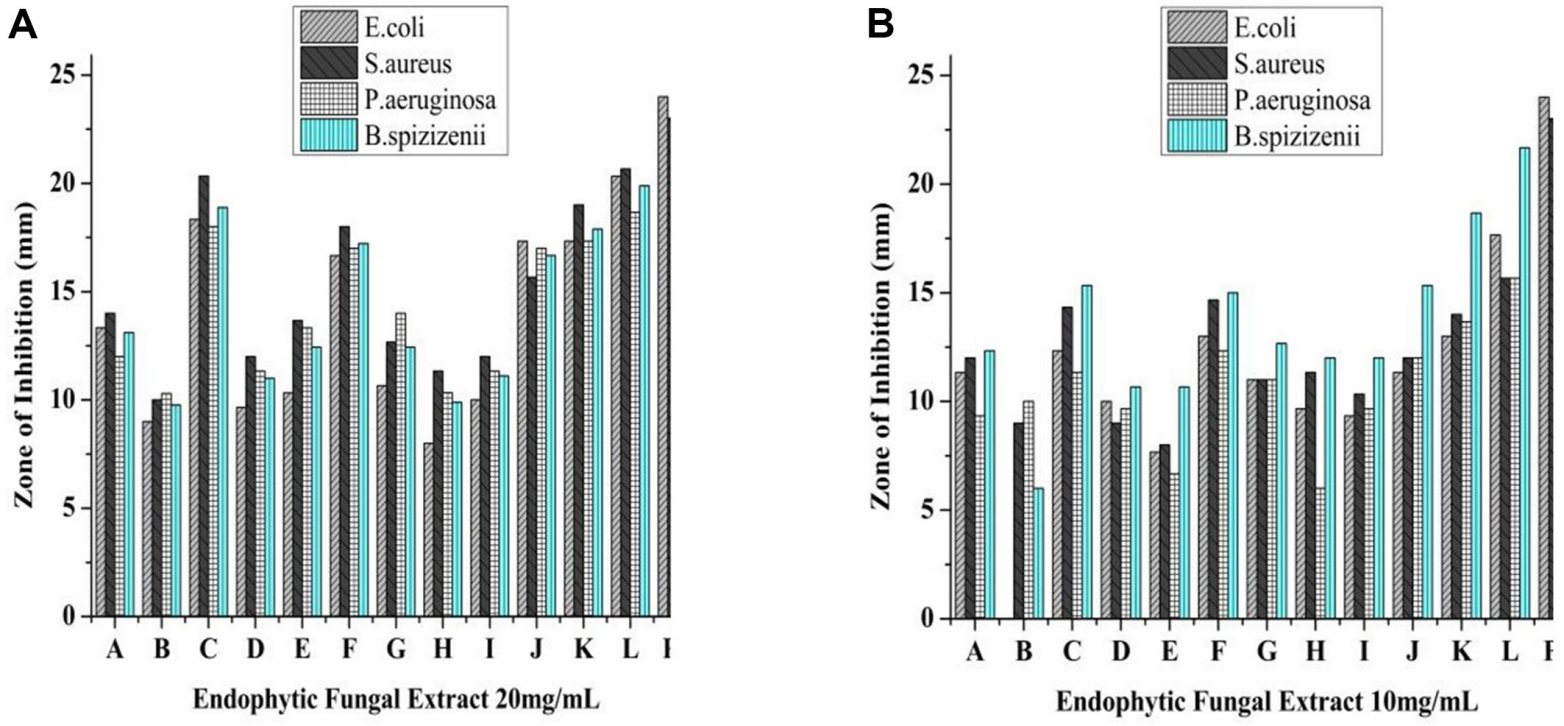
(A) Antibacterial activity of extracts at 20 mg/mL concentration, where A (SML2.1), B, (SML2.2), C (SML3), D (SML4.1), E (SML5), F (SML6.2), G (SMS1), H (SMS2), I (SMS3), J (SMS4), K (SMS6), L (SMF2) and P+ (Ciprofloxacin) and (SML = *S. marianum* leaf, SMS = *S. marianum* Stem and SMF = *S. marianum* fruit, while 2.1, 2.2, 3, 4.1, 5, 6, and 6.2 are the plate number). (B) Antibacterial activity of extracts at 10 mg/ml concentration, where A (SML2.1), B, (SML2.2), C (SML3), D (SML4.1), E (SML5), F (SML6.2), G (SMS1), H (SMS2), I (SMS3), J (SMS4), K (SMS6), L (SMF2) and P+ (Ciprofloxacin) and (SML = *S. marianum* leaf, SMS = *S. marianum* Stem and SMF = *S. marianum* fruit, while 2.1, 2.2, 3, 4.1, 5, 6, and 6.2 are the plate number).

All extracts exhibited antibacterial activity at a dose of 20 mg/ml but the highest activity was exhibited by *S. marianum* fruit (SMF2) that is 20.3 mm against *E. coli*, 18.66 mm against *P. aeruginosa*, 19 mm against *S. aureus*, and 22 mm against *B. spizizenii*. The other extracts like SML3, SML6.2 (*Silybum marianum* leaf) and SMS6 (*Silybum marianum* stem) also indicated good antibacterial activity while the other extracts exhibited marginal activity (Fig. 1A and 1B).

The IC_50_ values ranged from 0.73 to 9.44 mg/ml. SML2.2, SML3, SMS2, SMS6 and SMF2 extracts have IC_50_ values of 2.83, 0.82, 0.97, 0.73, and 1.83 mg/ml, respectively, against *E. coli*, whereas the others exhibited IC_50_ values greater than 4 mg/ml. In the case of *P. aeruginosa*, extracts of SML3, SMS2, SMS6 and SMF2 indicated IC_50_ values at 1.11, 2.90, 0.77 and 1.01 mg/ml, whereas the others all have a MIC above 4 mg/ml. SML3, SML5, SMS6, and SMF2 exhibited IC_50_ value between 1.47 to 2.53 mg/ml against *S. aureus*. Against *B. spizizenii*, SML3, SMS2, SMS6, and SMF2 indicated IC_50_ values between at 1.72 to 2.75 mg/ml (Table 1). SML3 and SMF2 extracts exhibited potential bactericidal activity against the tested pathogenic bacterial strains (*S. aureus*, *B. spizizenii*, *E. coli*, and *P. aeruginosa*) with complete inhibition of growth at 1.25 mg/ml while SML 2.2 controlled growth of selected bacterial pathogens at a concentration of a dose of 1.25 mg/ml. Other extracts exhibited MCB at concentration of more than 5 mg/ml.

### Antioxidant Activity of Fungal Endophytes

**Fig. 2:**
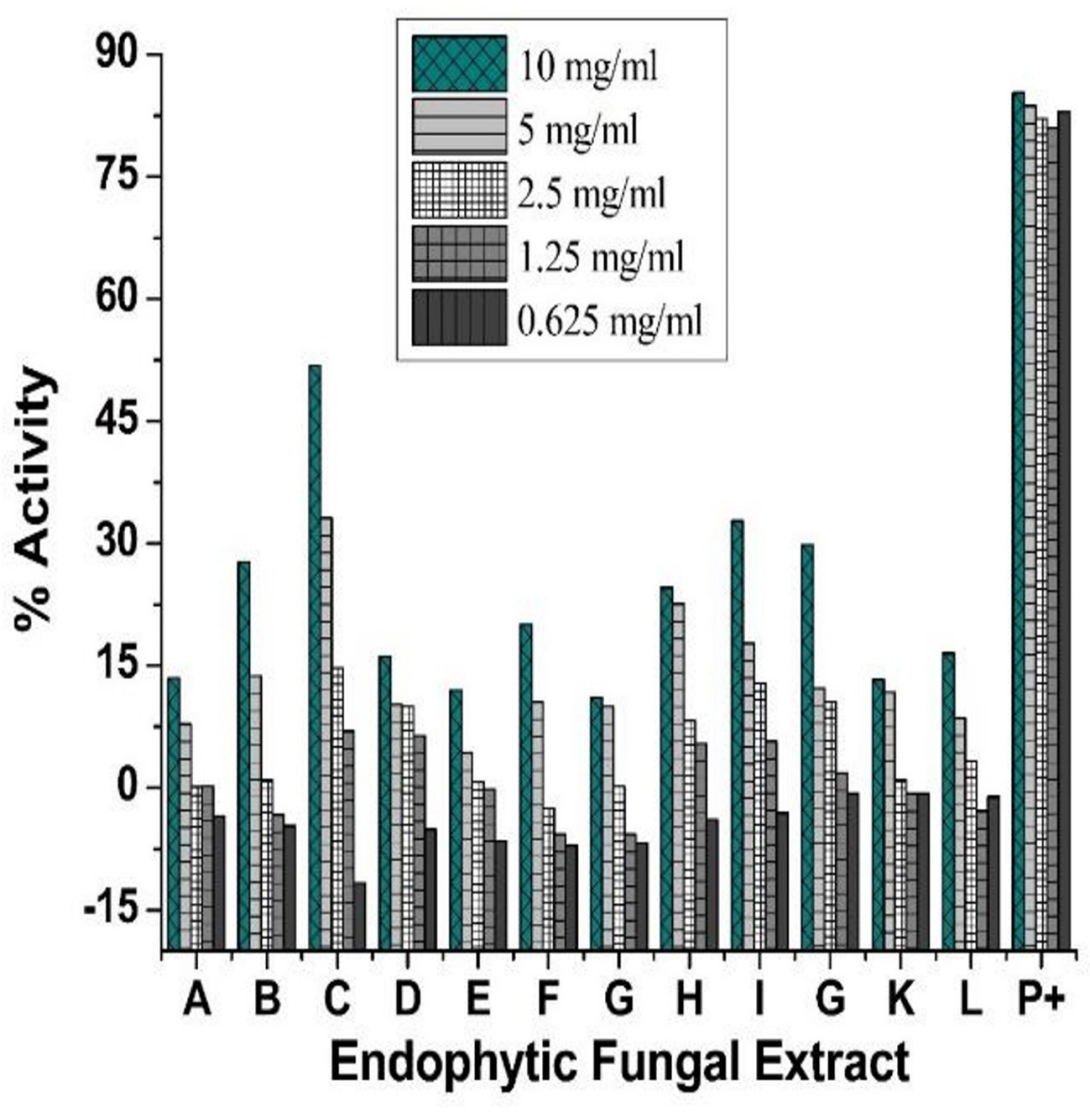
Antioxidant activity of various concentrations of endophytic fungal extracts, where A (SML2.1), B, (SML2.2), C (SML3), D (SML4.1), E (SML5), F (SML6.2), G (SMS1), H (SMS2), I (SMS3), J (SMS4), K (SMS6), L (SMF2) and P+ (ascorbic acid) while (SML = *S. marianum* leaf, SMS = *S. marianum* Stem and SMF = *S. marianum* fruit, while the numbers 2.1, 2.2, 3, 4.1, 5, 6, and 6.2 indicate the plate number).

During antioxidant assay greatest antioxidant activity was observed in crude extracts of SML3 and SMF2 as 51.82 and 32.77% at the quantity of 10 mg/ml. The other extracts indicated low antioxidant activity at the same concentration as compared to the ascorbic acid-based positive control (Fig. 2).

### Inhibition of Biofilm Formation

Extracts of endophytic fungi exhibited 50% inhibition of biofilms with the least efficacy of inhibition of biofilm caused by SMF2 and SML3 12.33 and 12.91 mg/ml against *P. aeruginosa* and 4.62 and 3.63 against *S. aureus*. Alternatively, SMS6, and SML6.2 also inhibited biofilm formation with 15.16 and 18.44 mg/ml against PA01 and 6.82 and 6.7 mg/ml against *S. aureus*. Other extracts were not able to significantly, inhibit the formation of biofilm (Table 2).

### Biocompatibility of Extracts

To assess the biocompatibility of extracts of cultures of endophytic fungi, an in vitro hemolytic assay was performed to measure damage to RBC when exposed to extracts. Extract of fungal isolate SMF2 exhibited 2.54%hemolytic activity at a quantity of 10 mg/ml in comparison to 83% of Triton, the positive control. Other extracts SML3, SML6.2, and SMS6 were also deemed to biocompatible and unlikely to cause adverse effects in vivo, since they exhibited less than 10% of hemolytic activity at all tested concentration. The hemolytic activities of extracts are very low, and we can say that these extracts have biocompatibility for further use (Table 3).

### Identification of Endophytic Fungi from *S. marianum*

**Fig. 3:**
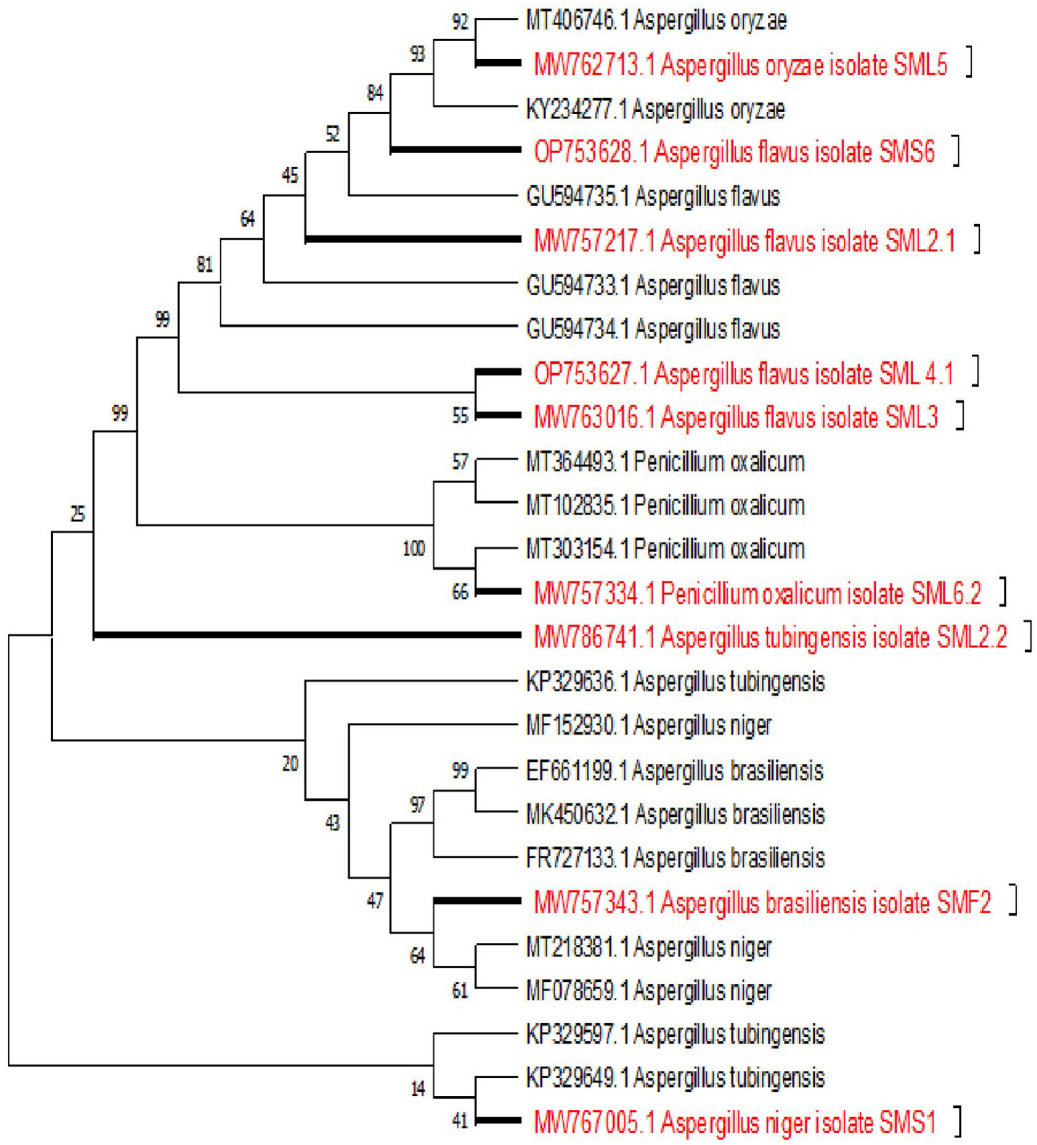
The phylogenetic tree indicating evolutionary history using the Neighbor-Joining method.

Based on the results of biological evaluation, biologically active nine fungal strains were subjected to molecular identification. These fungi were identified as *A. flavus* (SML2, MW757217), *A. tubigenesis* (SML2.2, MW763016), *A. flavus* (SML3, MW762713), *A. flavus* (SML4, MW762713), *A. oryzae* (SML5, MW767334), and *Penicillium oxalicum* (SML6.2, MW767005) from leaves of plant. While *A. niger* (SMS1, MW767005) and *A. flavus* (SMS6) were identified from stem of plant and *Aspergillus brasiliensis* (SMF2, MW757343) was isolated and identified from fruits of *S. marianum* (Table 4 and Fig. 3).

The evolutionary history was inferred using the Neighbor-Joining method [41]. The bootstrap consensus tree inferred from 1,000 replicates is taken to represent the evolutionary history of the taxa analyzed. Branches corresponding to partitions reproduced in less than 50% bootstrap replicates are collapsed. The percentage of replicate trees in which the associated taxa clustered together in the bootstrap test (1,000 replicates) are shown next to the branches. The evolutionary distances were computed using the Kimura 2-parameter method in the units of the number of base substitutions per site. This analysis involved 26 nucleotide sequences. All ambiguous positions were removed for each sequence pair (pairwise deletion option). There was a total of 511 positions in the final dataset. Evolutionary analyses were conducted in MEGA X [44].

### Isolation and Structural Characterization of Bioactive Compounds from Fungus SMS6

Preparative chromatographic techniques using silica gel 60 or Sephadex LH 20 were used to purify two compounds from the crude ethyl acetate extract from the above-mentioned fungus. The molecular formula of compound 1 was determined as C_6_H_6_O_4_ based on the [M+Na]^+^ peak at *m/z* = 165.0162 (calcd 165.0164 for C_6_H_6_NaO_4_) in the ESI spectrum. The ^1^H NMR spectrum showed 3 singlet signals at δ_H_ 7.50 (1H), 6.50 (1H), and 4.41 (2H) ppm, respectively, whereas the ^13^C NMR spectrum revealed in accordance to mass data the presence of 6 carbon signals. All spectral data are identical to those published for kojic acid (lit), a common secondary metabolite of *Aspergillus* species.

**Fig. 4:**
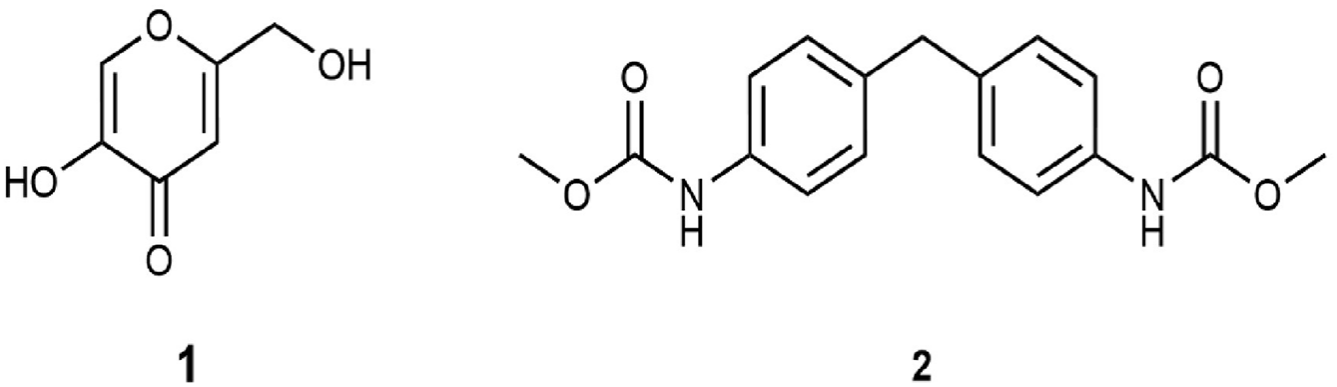
Structural formulae of kojic acid (1) and carbamic acid (methylene-4, 1-phenylene) bis-dimethyl ester (2).

The molecular formula of compound 2 was determined as C_17_H_18_N_2_O_4_ based on the [M+Na]^+^ peak at *m/z* = 337.1164 (calcd 337.1164 for C_17_H_18_N_2_NaO_4_) in the ESI spectrum. The ^1^H NMR spectrum showed the characteristic doubletic pattern of a *para*-substituted benzene at δ_H_ 7.28 and 7.10 ppm. Combined analysis of ^1^H, ^13^C and HSQC spectra revealed also the presence of a methoxyl group at δ_H_/δ_C_ 3.76/52.3 ppm, a methylene group at δ_H_/δ_C_ 3.88/40.6 ppm and one exchangeable proton (NH) at at δ_H_ 6.53 ppm. In addition, quaternary carbons were detected at δ_C_ 135.9, 136.4, and 154.0 ppm. The HMBC spectrum connected the methoxyl group to the carbon at δ_C_ 154.0 ppm, whereas the other two quaternary carbons could be assigned to the benzene ring. Therefore, the structure of 2 was established as carbamic acid (methylene-4, 1-phenylene) bis-dimethyl ester previously already isolated from *Magnolia kachirachirai* (Kaneh. & Yamam.) (Fig. 4). Dandy (Magnoliaceae) [43]. The MR and mass spectra of the isolated compounds are provided in the Supplement file.

### Bioactivity of the Purified Compounds

**Radical scavenging activity.** The identified compounds kojic acid (1) and carbamic acid (methylene-4, 1-phenylene) bis-dimethyl ester (**2**) indicated significant antioxidant activity. Dose-dependent response was noticed for both compounds however kojic acid exhibited lower EC_50_ value of 99.3 μg/ml whilst carbamic acid,(methylene-4, 1-phenylene) bis- dimethyl (2) exhibited an EC_50_ value of 228 μg/ml (Table 5).

**Antibacterial activity.** Antibacterial activity indicated that both the compounds were not able to inhibit the growth of *Lactobacillus lactis* and *E. coli* strains at the concentrations tested in the range of 0.0156–2.0 μg/ml while the positive control was able to produce zone of inhibition of 23–24 mm at various concentrations.

**Fig. 5:**
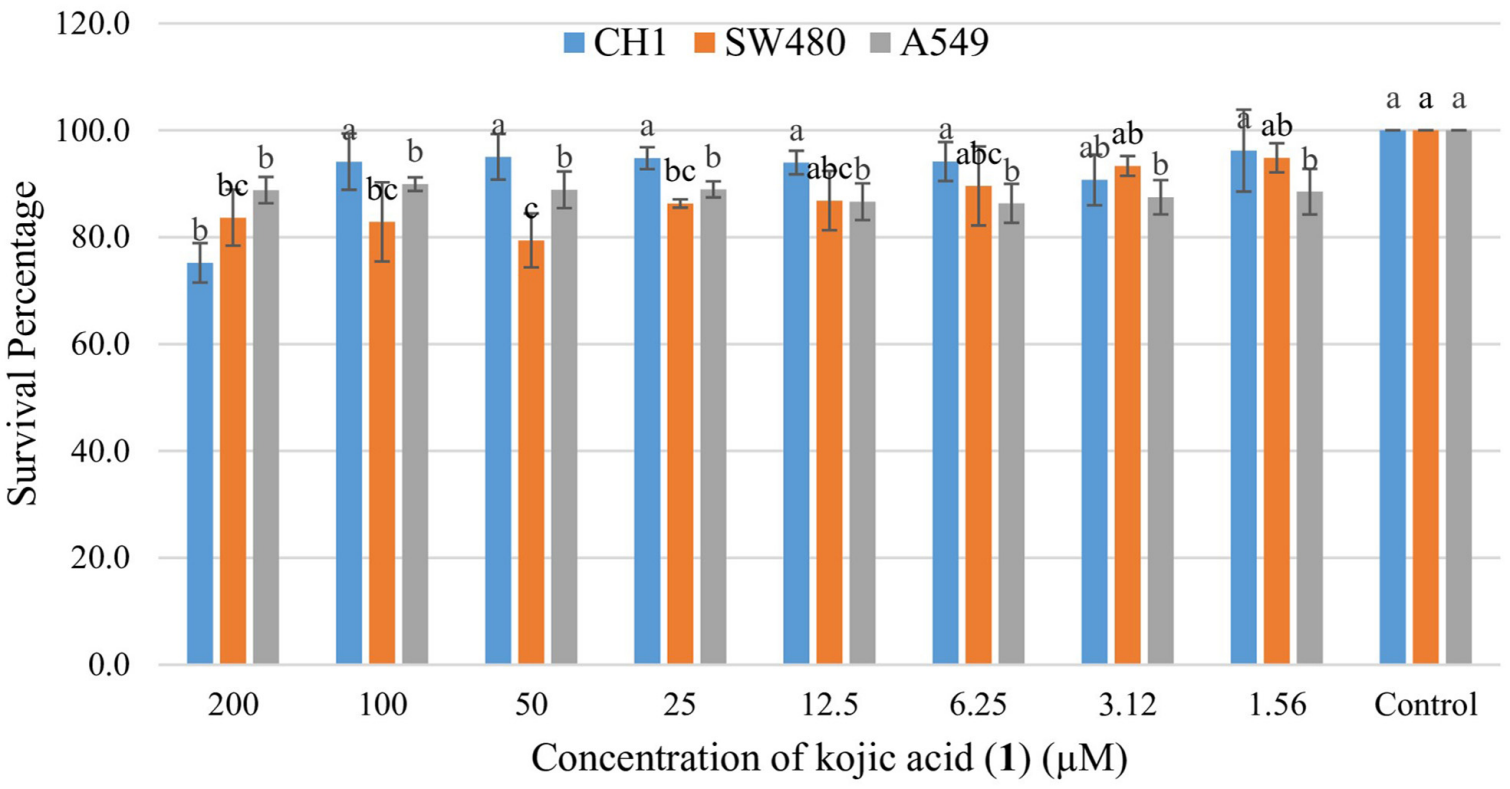
Anticancer activity of Kojic acid (1) against tested cancer cell lines.

**Anticancer activity.** Kojic acid (1) exhibited significant antioxidant activity and hence was proceeded for anticancer activity assays against three cell lines. Kojic acid exhibited weak activity against the tested cancer cell lines with 75% survival rate for CH1 cells at 200 μg/ml followed by 89% and 88% survival of SW480 and A549 cells respectively (Fig. 5).

## Discussion

The investigation of bioactive potentials of crude ethyl acetate extracts of various endophytic fungi for antibacterial, antibiofilm, antioxidant, and haemolysis activities indicated moderate bioactive potential of all of the isolated species. The extracts obtained from SMS6, SML3, and SMF2 exhibited significant activity in all assays. The sample SMS6 (*A. flavus*) was further selected for purification of components responsible for bioactivity, based on the better bioactive potential and being most biocompatible. The results of antibacterial activity are consistent with previous studies, which also reported antibacterial activities of endophytic fungi against both Gram-negative and Gram-positive bacteria [45, 46]. The IC_50_ values of crude extract indicated potencies of extracts, expressed as IC_50_, ranged from 0.47–9.44 mg/ml. The extracts SMS6 and SML3 exhibited similar IC_50_ values with little variation in a range of 0.82 to 1.72 mg/ml against selected bacterial strains of both Gram positive and Gram-negative bacteria. The antibacterial activity varied from the results in previous study [47] who reported MIC values fungal extracts lower than 0.125 mg/ml. This can be attributed to the fact, that fungi isolated from different plants, even from same plants of different geographical areas and from different parts of the same plant might vary in secondary metabolite composition and hence bioactivity. Different factors are involved in the production of secondary metabolites, which can affect the bioactivity of same specie isolated from different parts of same plant. *Moharram* *et al*. (2015) indicated that the same fungi isolated from same plant being cultured on same media and by adopting same procedure were indicating different levels of kojic acid production [48]. Similarly *Kusari* *et al*.(2012) explained that endophyte-endophyte interspecies crosstalk can play an important role in gene switching on and off during the course of stay within a specific organism [49]. Further studies indicate that different factors play important role in the production of secondary metabolites from endophytic fungi [50]. Study elaborates that transcription factors play important role in the secondary metabolite production, and classified transcription factors into two large groups that are narrow domain transcription factors (NDTFs) and broad domain transcription factors (BDTFs). These factors work in association to perform through specific biochemical cascade reactions, including methylation, phosphorylation, and acetylation. These reactions are essential for activating the silent clustered genes associated with specific secondary metabolites that associated with cellular metabolisms of particular organ, growth stages, or environmental conditions. Significant biofilm (90–80%) reductions were observed in biofilm produced by *Pseudomonas aeruginosa* (PAO1) at dosages of 20 mg/ml of either SMF2 and SML3, which are contrary to findings reported previously [51, 52] where 54.41% reduction in biofilm formation by *P. aeruginosa* was reported.

The assessed radical scavenging activities indicated a maximum of 51.8% antioxidant potential of SML3 (*A. flavus*). Similarly, fungal endophytes exhibited excellent properties to scavenge the reactive oxygen species (ROS) and/or superoxide radicals, in particular, polyphenols, which are potent inhibitors of oxidation [53]. Presumably, phenolic compounds and/or anthraquinones might be present in the studied extracts. Our investigations also revealed significant antioxidant activity of kojic acid (1) making it a useful ingredient for cosmetics [54].

Regarding hemolysis, hemolytic effects less than 10% are considered as safe [37]. The crude extracts of SML3, SML6.2, SMS4, SMS6 and SMF2 were found compatible to red blood cells during initial experimentation, suggesting that these strains can further be considered as potentials for development of therapeutic agents.

Contrary to previous reports, kojic acid (1) exhibited no antibacterial activity against the tested bacterial strains. Another study supports our result that kojic acid (1) exhibited no evident antibacterial activity, however its ester derivatives indicated activities against bacterial strains [55]. Our study reports weak activity of kojic acid (1) against cancer cell lines, namely MCF-7 and MDA-MB-231 breast cancer cell line, A375 human malignant melanoma cell lines HEPG2 hepatocellular carcinoma cell line, VC-8-BRCA and VC-8, (ovarian cancer cell lines) and caco-2 colon cancer cell line. The possible mechanism adopted by these derivatives was necrosis and apoptosis [56]. A number of studies reported a positive correlation of antioxidants in cancer therapy [57]. Levels of intracellular and extracellular ROS, are generally raised in cancer cells [58]. The elevated level of ROS in cancer cells is double-edged, having protumorigenic or cytotoxic effects, depending on concentration. At levels above a cytotoxic threshold, ROS cause cancer cell death via DNA, lipid, and protein damage. Below this cytotoxic threshold but above the level found in non-cancerous cells, ROS stimulates tumor growth and progression [60]. The second compound was carbamic acid, (methylene-4, 1-phenylene) bis-dimethyl ester (2). This compound has also been purified from the ethyl acetate extract of the endophytic fungus *Penicillium citrinum* HL-5126 isolated from the mangrove *Bruguiera sexangula* var. *rhynchopetala* W.C.Ko (syn. of *Bruguiera rhynchopetala*)[61]. Compound 2 also indicated antioxidant effects, but its antibacterial effects were not determined against selected bacterial strains.

## Conclusion

The findings of this study reveal the potential of endophytic fungi from *S. marianum* to produce compounds with antimicrobial, antioxidant, and antibiofilm properties. Fungal endophytes from *S. marianum* may serve as an alternative source of bioactive substances. The ethyl acetate extract exhibited high antibacterial activity against *S. aureus*, *Salmonella typhimurium*, *P. aeruginosa*, *Bacillus spizizenii*, *P. aeruginosa* PAO1 and *S. aureus* strains was used to determine antibiofilm activity and *A. flavus* and *A. brasiliensis* exhibited good antibiofilm as well as antioxidant activities. The compounds isolated from the endophytic fungi further confirm the bioactive potential of fungi. Our future studies will be focused on determination of mechanism of action of purified compounds.

## Supplemental Materials

Supplementary data for this paper are available on-line only at http://jmb.or.kr.



## Figures and Tables

**Table 1:** IC_50_ values (mg/ml) of crude fungal extracts against selected bacterial strains.

S. No	Extract	IC_50_ values of fungal extracts			
		*E. coli*	*P. aeruginosa*	*S. aureus*	*B. spizizenii*
1	SML2.1	4.56	7.33	5.68	6.17
2	SML2.2	2.83	4.06	4.09	4.37
3	SML3	0.82	1.11	1.47	1.72
4	SML4.1	9.44	4.84	7.54	5.49
5	SML5	6.08	5.48	5.24	6.49
6	SML6.2	5.45	5.32	5.09	5.62
7	SMS1	5.55	5.57	5.53	6.27
8	SMS2	0.97	2.90	1.93	2.20
9	SMS3	8.20	8.17	7.62	8.88
10	SMS4	4.61	4.47	4.51	4.63
11	SMS6	0.73	0.77	2.44	2.45
12	SMF2	1.83	1.01	2.53	2.75

SML = *S. marianum* leaf, SMS = *S. marianum* Stem and SMF = *S. marianum* fruit, the numbers 1, 2, 2.1, 2.2, 3, 4.1, 5, 6, and 6.2 indicate the plate number.

**Table 2:** Inhibition of biofilm by endophytic fungal extracts.

S. No	Extract Name	*PAO1*	*S. aureus*
1	SML2.1	27.83	10.71
2	SML2.2	29.51	21.8
3	SML3	12.91	3.63
4	SML4.1	92.91	6.16
5	SML5	106.21	10.93
6	SML6.2	18.44	6.7
7	SMS1	43.18	4.58
8	SMS2	17.79	287.6
9	SMS3	18.68	7.75
10	SMS4	193.36	7.56
11	SMS6	15.16	6.82
12	SMF2	12.33	4.02

SML = *Silybum marianum* leaf, SMS = *Silybum marianum* Stem and SMF = *Silybum marianum* fruit, while 1, 2, 2.1, 2.2, 3, 4.1, 5, 6, and 6.2 are the plate numbers). The IC_50_ values are given in mg/ml.

**Table 3:** Hemolytic activity (Percentage) of endophytic fungal crude extracts.

Extracts		Concentration [mg/ml]				
		10	5	2.5	1.25	0.625
Leaf isolates	SML2.1	28.27	26.2	19.8	18.99	18.78
	SML2.2	19.43	22.77	17.94	13.98	17.74
	SML3	9.7	9.6	6.16	5.7	1.7
	SML4.1	29.29	29.48	25.89	20.44	19.04
	SML5	30.3	26.52	19.86	19.12	11.01
	SML6.2	7.9	2.3	1.2	0.3	0.7
Stem isolates	SMS1	19.06	18.7	13.9	12.33	12.04
	SMS2	28.50	27.61	26.50	18.7	13.9
	SMS3	3.50	3.53	1.50	1.18	0.78
	SMS4	4.53	3.60	1.63	1.38	0.39
	SMS6	4.93	3.3	1.4	1.18	0.78
Fruit isolates	SMF2	2.54	2.54	1.38	0.39	0.21
	Triton X	83.53	69.9	59.6	59.6	34.9

SML = *S. marianum* leaf, SMS = *S. marianum* Stem and SMF = *S. marianum* fruit, Triton X = control, while 2.1, 2.2, 3, 4.1, 5, 6, and 6.2 are the plate numbers.

**Table 4:** Identification of endophytic fungi on the basis of sequencing results.

S. No	Isolates	Source	18S r RNA amplified region length	Similarity [%]	NCBI accession number
1	SML2.1	Leaf	618 bps	*99.83 Aspergillus flavus*	MW757217
2	SML2.2	Leaf	618 bps	*99.35 Aspergillus tubigenesis*	MW786741
3	SML3	Leaf	618 bps	*98.86 Aspergillus flavus*	MW763016
4	SML4.1	Leaf	618bps	*98.76 Aspergillus flavus*	OP753627
5	SML5	Leaf	618 bps	*98.67 Aspergillus oryzae*	MW762713
6	SML6.2	Stem	618 bps	*99.46 Penicillium oxalicum*	MW757334
7	SMS1	Stem	618 bps	*95.46 Aspergillus niger*	MW767005
8	SMS6	Stem	618 bps	*96.56 Aspergillus flavus*	OP753628
9	SMF2	Fruit	618 bps	*99.29 Aspergillus brasiliensis*	MW757343

SML = *S. marianum* leaf, SMS = *S. marianum* Stem and SMF = *S. marianum* fruit, while 2.1, 2.2, 3, 4.1, 5, 6, and 6.2 are the plate numbers.

**Table 5:** Antioxidant activity of purified compounds.

Compounds	EC_50_ [μg/ml]
Kojic acid (**1**)	99.5
Carbamic acid (methylene-4, 1-phenylene) bis-dimethyl ester (**2**)	228
