# Supplementary material for: HPLC, NMR Based Characterization, Antioxidant and Anticancer Activities of Chemical Constituents from Therapeutically Active Fungal Endophytes
Source: J Microbiol Biotechnol. 2024 May 6;34(7):1452–63. doi: 10.4014/jmb.2403.03036 (PMC11294646; doi:10.4014/jmb.2403.03036)
Supplement: Supplementary file 1 [file jmb-34-7-1452-supple.pdf]

J. Microbiol. Biotechnol. 2024. 34(7): 1452–1463
https://doi.org/10.4014/jmb.2403.03036

Supplementary Figures

HPLC, NMR based characterization, antioxidant and anticancer activities of chemical constituents from therapeutically active fungal endophytes

Waqas Hussain Shah[1,4], Wajiha Khan[1*], Sobia Nisa[2*], Michael H.J. Barfuss [3], Johann Schinnerl [3], Markus Bacher[4,] Karin Vetschera[3], Ashraf Ali[5,6], Hiba-Allah Nafidi[7], Yousef A. Bin Jardan[8], John P. Giesy[9,10,11,12,13]

[1]Department of Biotechnology, COMSATS University Islamabad, Abbottabad Campus
[2]Department of Microbiology, The University of Haripur, Haripur 22620, Pakistan
[3]Department of Botany and Biodiversity Research, University of Vienna, Rennweg 14, 1030 Vienna, Austria [4]Department of Chemistry, Institute of Chemistry of Renewable Resources, University of Natural Resources and Life Sciences (BOKU), Tulln 3430, Austria
[5]Department of Chemistry, Faculty of Physical and Applied Sciences, The University of Haripur, Haripur 22620, Pakistan [6]School of Chemistry and Chemical Engineering, Henan University of Technology, Zhengzhou 450001, P.R. China [7]Department of Food Science, Faculty of Agricultural and Food Sciences, Laval University, 2325 Quebec City, QC G1V 0A6, Canada [8]Department of Pharmaceutics, College of Pharmacy, King Saud University, P.O. Box 11451, Riyadh, Saudi Arabia [9]Toxicology Centre, University of Saskatchewan, Saskatoon, SK S7N 5B3 [10]Department of Veterinary Biomedical Sciences, University of Saskatchewan, Saskatoon, SK S7N 5B4, Canada [11]Department of Integrative Biology, Michigan State University, East Lansing, MI 48824, USA
[12]Department of Environmental Sciences, Baylor University, Waco, TX 76706, USA
[13]Department of Clinical Laboratory Sciences, College of Applied Medical Sciences, King Saud University, P.O. Box 10219, Riyah, 11433, Saudi Arabia

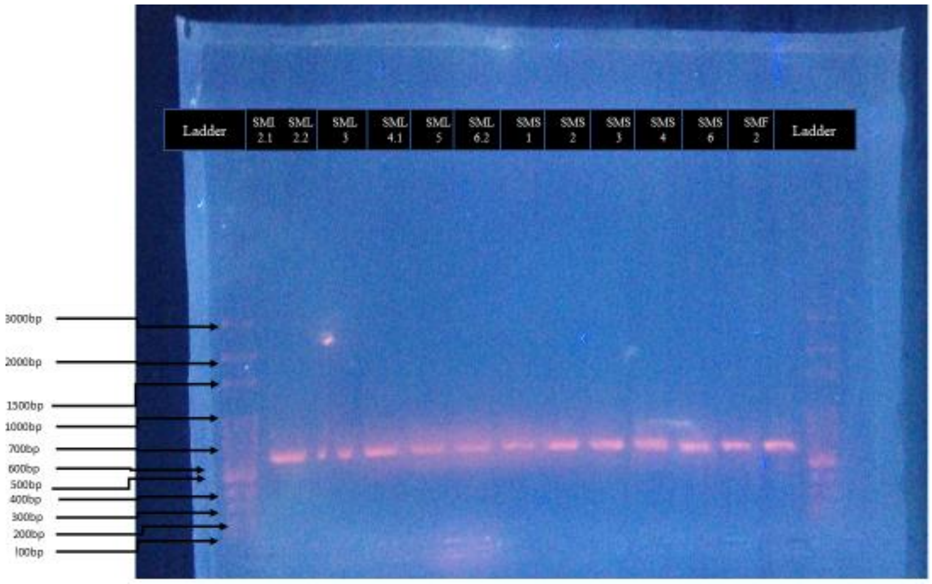

Supplementary Fig S1. Polymerase chain reaction of endophytic fungi. Both corners contain a ladder of (100-3000 bps).

The product size of the primer was 618 bps.

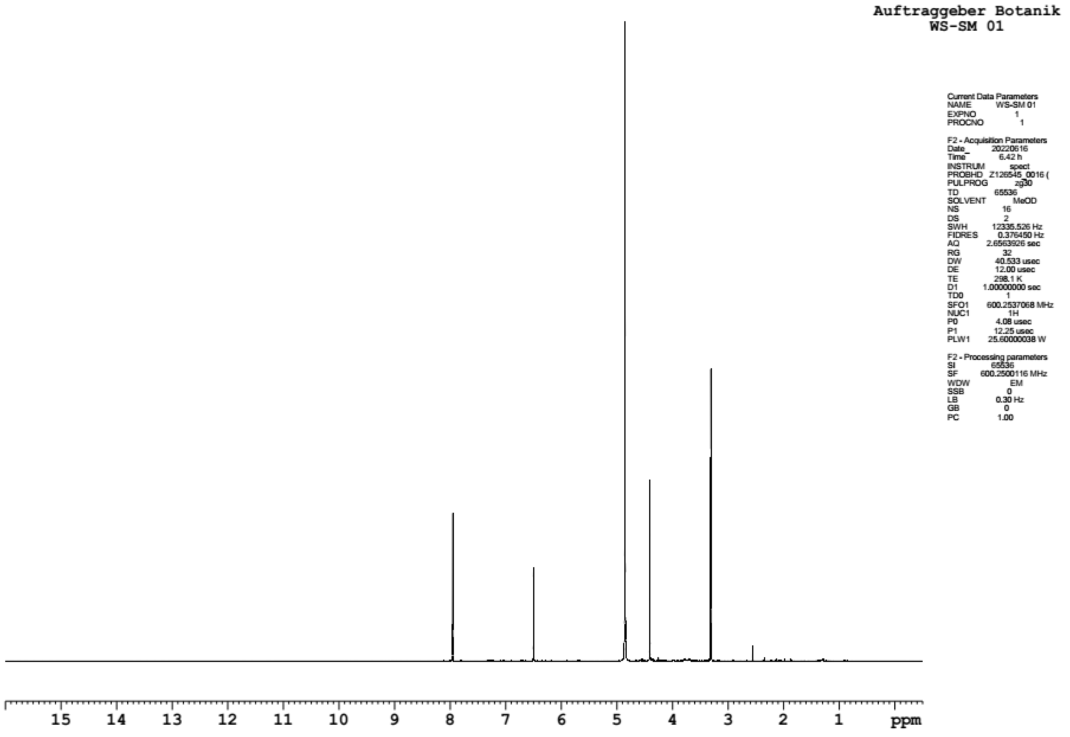

Supplementary Fig. S2. ^{1}H Spectrum of kojic acid.

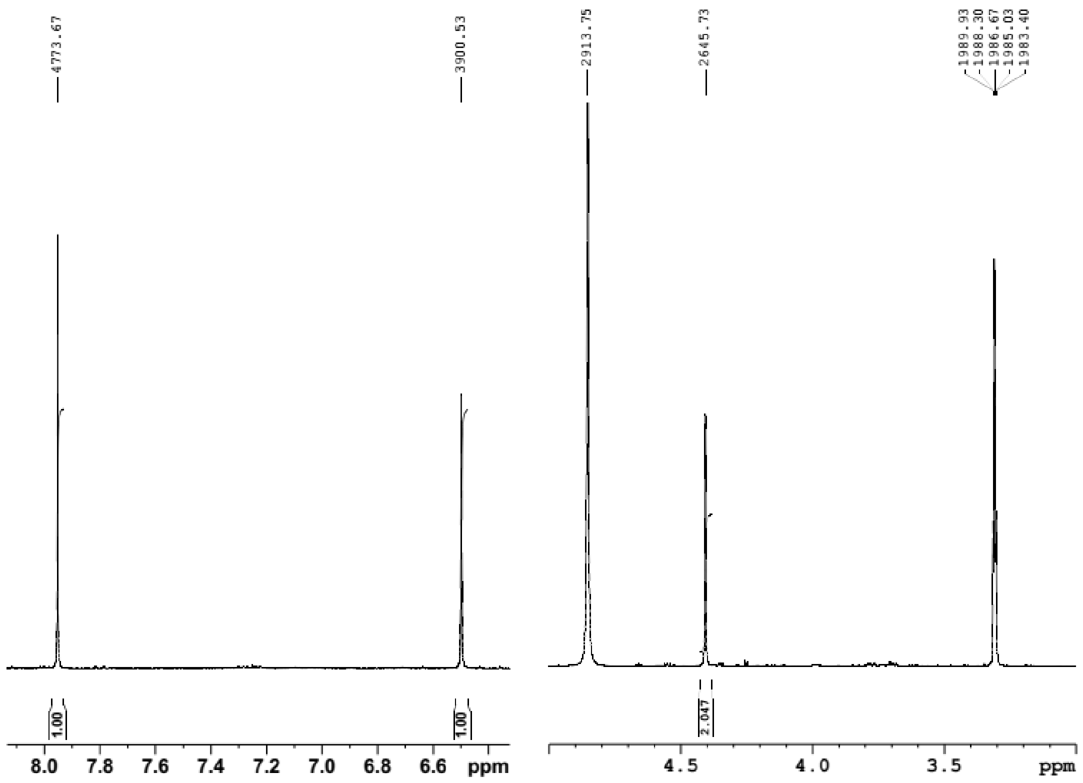

Supplementary Fig. S3. ^{1}H Spectrum of kojic acid, expanded regions.

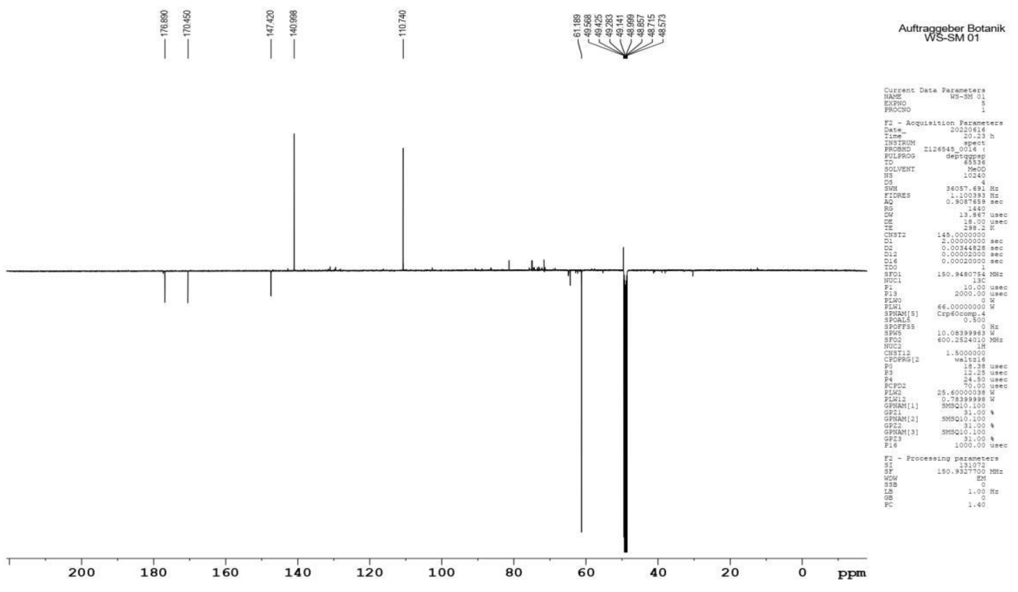

Supplementary Fig. S4.^{13}C jmod spectrum of kojic acid.

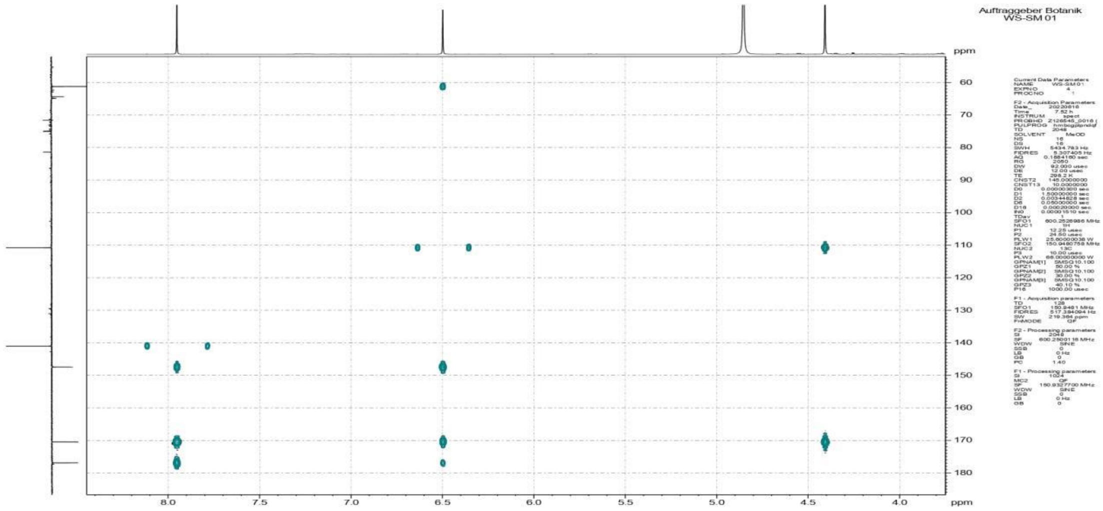

Supplementary Fig. S5. HMBC (Heteronuclear Multiple Bond Correlation) spectrum of kojic acid.

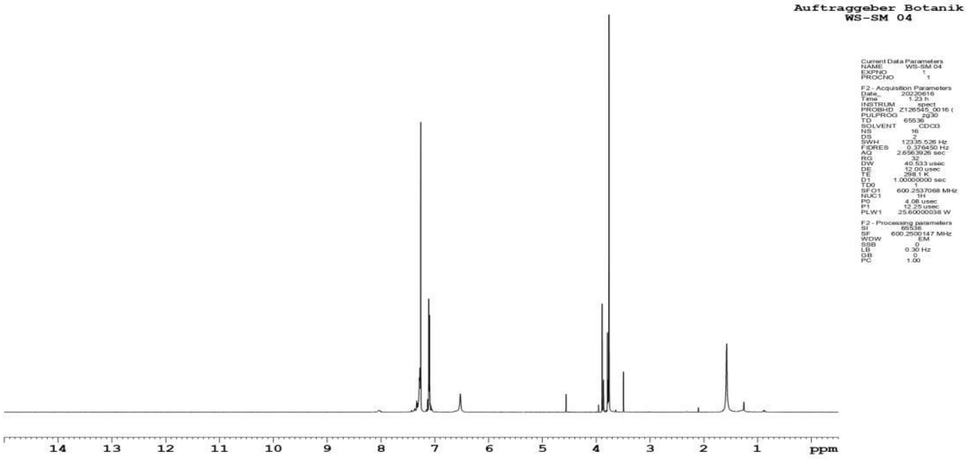

Supplementary Fig. S6: ^{1}H Spectrum of compound 2 carbamic acid (methylene-4, 1-phenylene) bis-dimethyl ester.

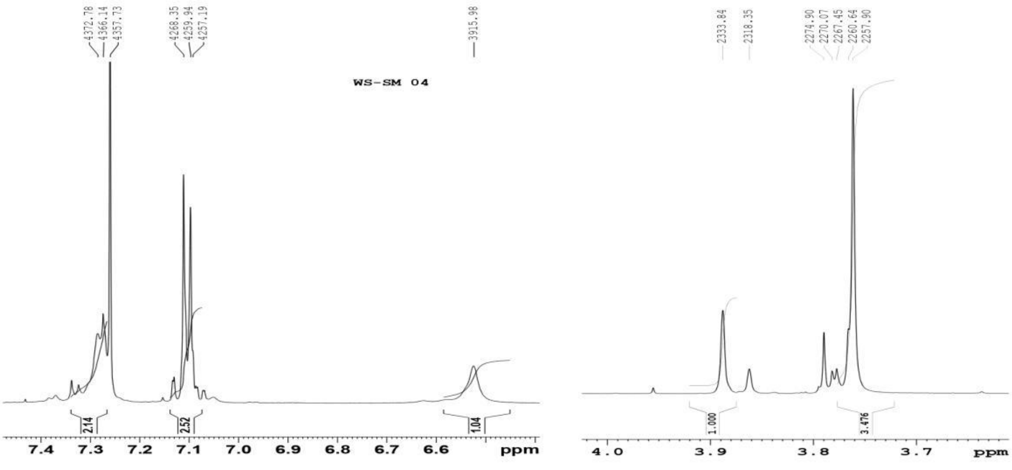

Supplementary Fig. S7: ^{1}H Spectrum of compound 2 carbamic acid (methylene-4, 1-phenylene) bis-dimethyl ester, expanded regions.

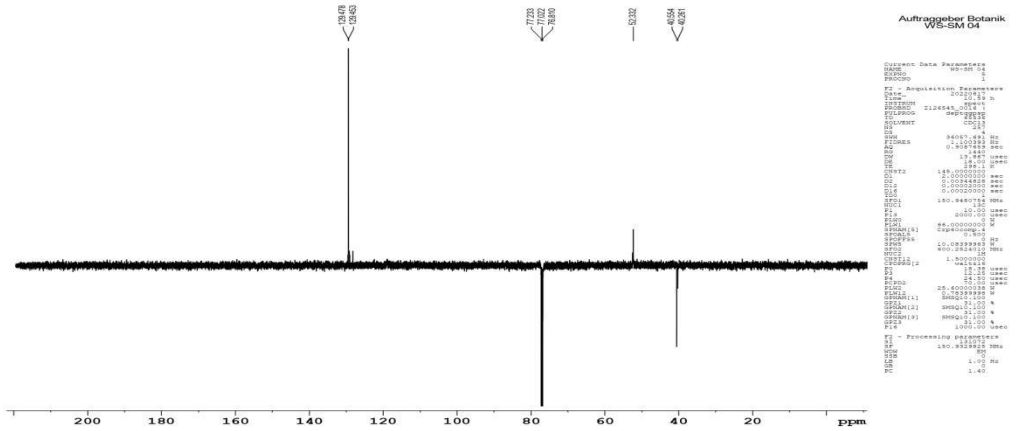

Supplementary Fig S8. ^{13}C jmod spectrum of carbamic acid.

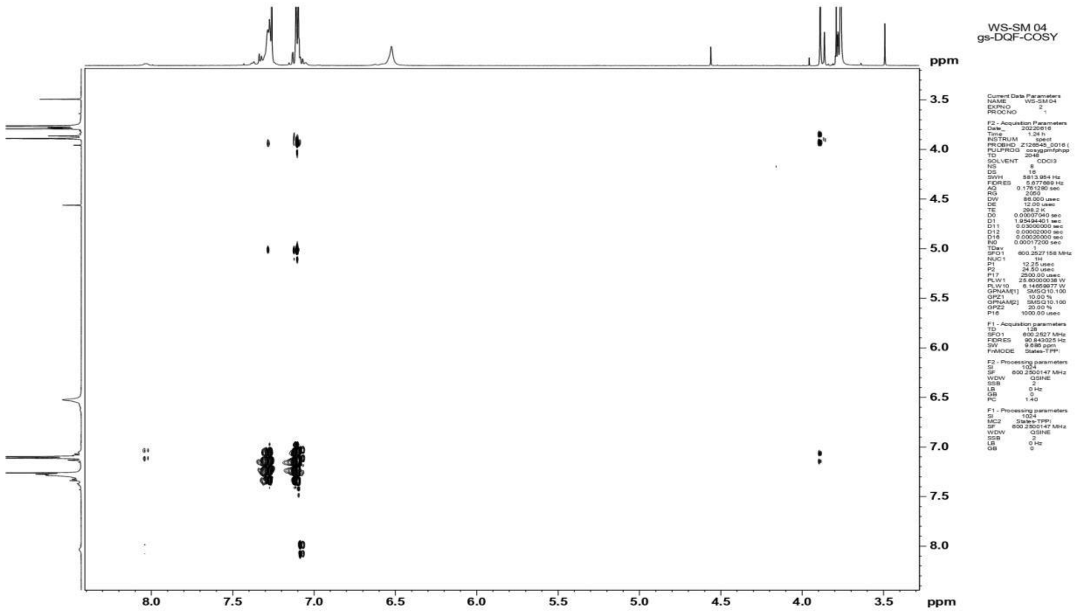

Supplementary Fig. S9. COSY spectrum of carbamic acid (methylene-4, 1-phenylene) bis-dimethyl ester.

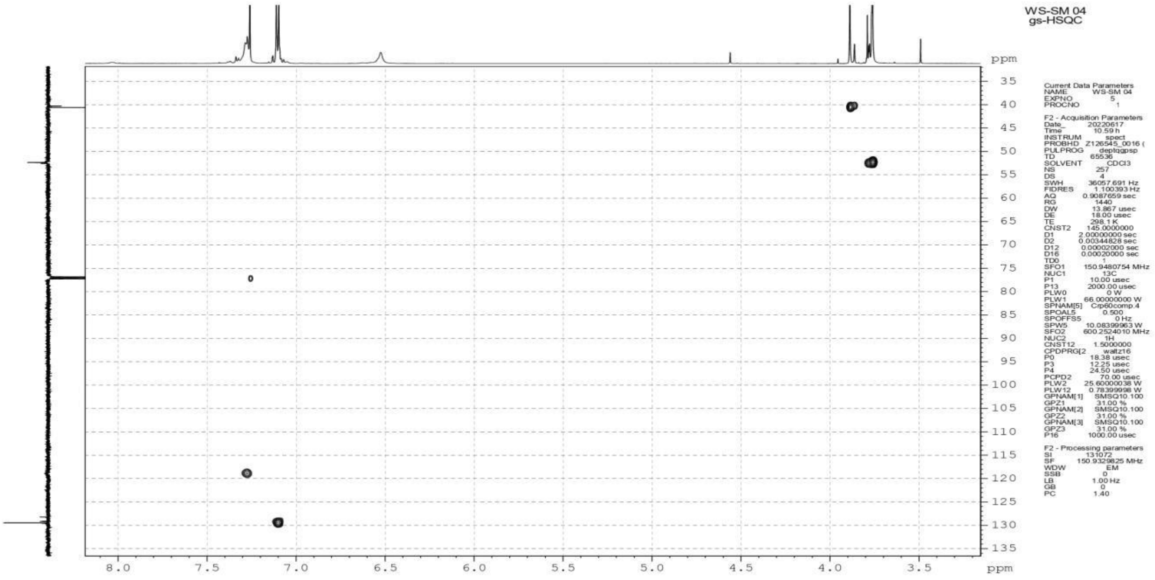

Supplementary Fig. S10. HSQC spectrum of carbamic acid (methylene-4, 1-phenylene) bis-dimethyl ester.

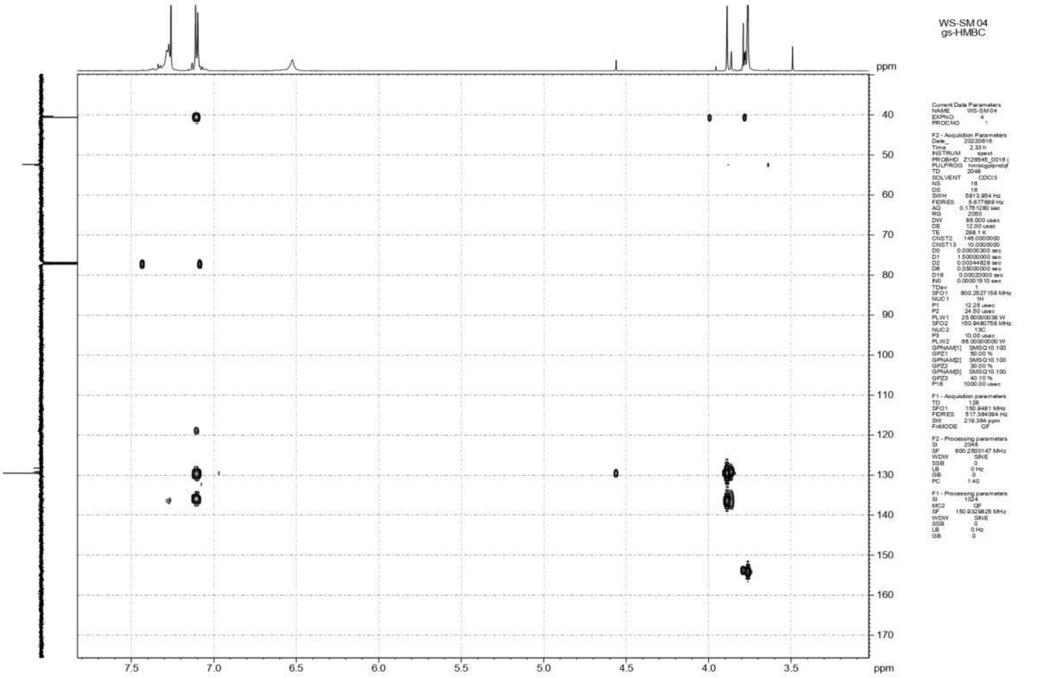

Supplementary Fig. S11. HMBC (Heteronuclear Multiple Bond Correlation) spectrum of carbamic acid (methylene-4, 1-phenylene) bis-dimethyl ester.

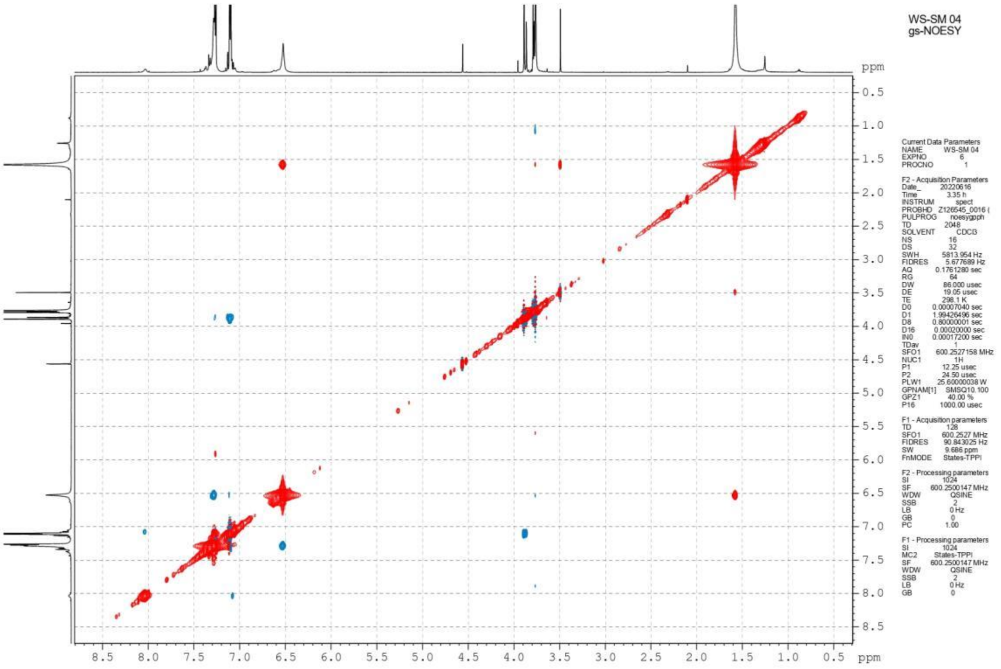

Supplementary Fig. S12. NOESY(Nuclear Overhauser Effect Spectroscopy) spectrum of carbamic acid (methylene-4, 1-phenylene) bis-dimethyl ester

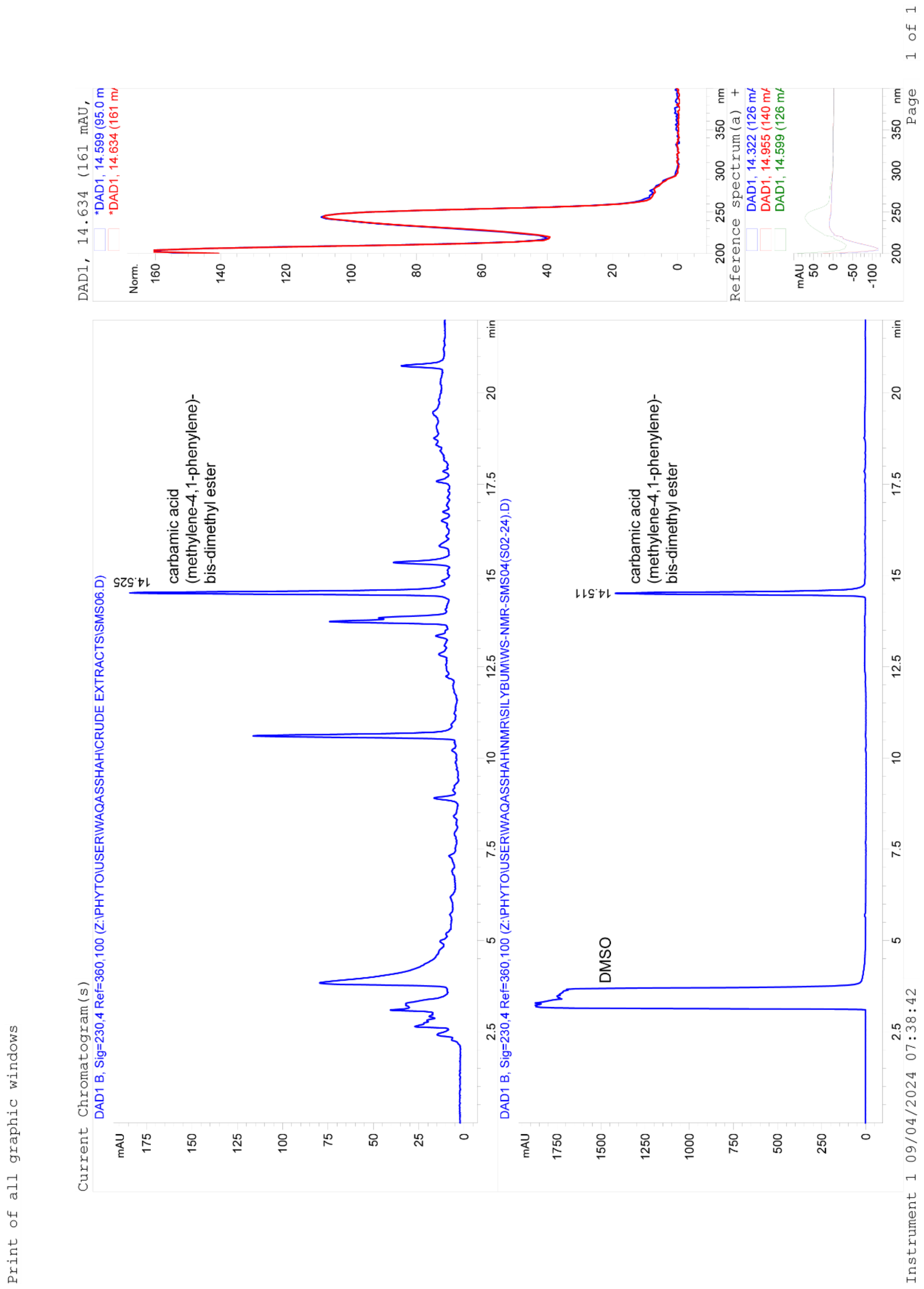

Supplementary Fig. S13. HPLC-UV-DAD chromatogram of the crude extract and the purified carbamic acid (methylene-4, 1-phenylene) bis-dimethyl ester.
